# Could probiotics protect against human toxicity caused by polystyrene nanoplastics and microplastics?

**DOI:** 10.3389/fnut.2023.1186724

**Published:** 2023-07-10

**Authors:** Javad Bazeli, Zarrin Banikazemi, Michael R. Hamblin, Reza Sharafati Chaleshtori

**Affiliations:** ^1^Department of Medical Emergencies, School of Nursing, Social Development and Health Promotion Research Center, Gonabad University of Medical Sciences, Gonabad, Iran; ^2^Research Center for Biochemistry and Nutrition in Metabolic Diseases, Institute for Basic Sciences, Kashan University of Medical Sciences, Kashan, Iran; ^3^Student Research Committee, Kashan University of Medical Sciences, Kashan, Iran; ^4^Laser Research Centre, University of Johannesburg, Doornfontein, South Africa; ^5^Social Determinants of Health (SDH) Research Center, Kashan University of Medical Sciences, Kashan, Iran

**Keywords:** probiotics, nanoplastics, microplastics, polystyrene, toxicity, gut microflora

## Abstract

Nanoplastics (NPs) and microplastics (MPs) made of polystyrene (PS) can be toxic to humans, especially by ingestion of plastic particles. These substances are often introduced into the gastrointestinal tract, where they can cause several adverse effects, including disturbances in intestinal flora, mutagenicity, cytotoxicity, reproductive toxicity, neurotoxicity, and exacerbated oxidative stress. Although there are widespread reports of the protective effects of probiotics on the harm caused by chemical contaminants, limited information is available on how these organisms may protect against PS toxicity in either humans or animals. The protective effects of probiotics can be seen in organs, such as the gastrointestinal tract, reproductive tract, and even the brain. It has been shown that both MPs and NPs could induce microbial dysbiosis in the gut, nose and lungs, and probiotic bacteria could be considered for both prevention and treatment. Furthermore, the improvement in gut dysbiosis and intestinal leakage after probiotics consumption may reduce inflammatory biomarkers and avoid unnecessary activation of the immune system. Herein, we show probiotics may overcome the toxicity of polystyrene nanoplastics and microplastics in humans, although some studies are required before any clinical recommendations can be made.

## 1. Introduction

The global use of plastics is increasing annually, and it has been predicted that plastic manufacturing may rise from 368 million tons in 2019 to 33 billion tons by 2050 (1, 2).

The widespread use of plastics in fields such as industrial production, medical manufacturing, construction, and agriculture can be attributed to their unique properties, such as chemical stability, light weight, wear resistance, and corrosion resistance. The accumulation of plastic aggregates highly resistant to degradation is an important global environmental issue because of their stable crystal structure and high molecular weight (2). Plastic particles smaller than 5 mm are defined as microplastics (MPs) while those smaller than 0.1 μm as nanoplastics (NPs) (2).

Polystyrene (PS) is a synthetic aromatic hydrocarbon polymer formed by polymerizing the styrene monomer (vinylbenzene), which is synthesized from benzene and ethylene followed by dehydrogenation of ethylbenzene. PS is a thermoplastic polymer with good transparency, long-lasting stability, and is easy to paint (3). It is a solid material extensively used to fabricate consumer products like toys, CDs, and toothbrushes, as well as to make Styrofoam. PS has limited elasticity and is melt-formed or expanded. Styrofoam has been extensively employed to fabricate food containers such as plates, trays, and cups, and is also used in a variety of packaging products, clips, toys, and office equipment (4).

PS MPs can be formed in the environment by photooxidative and mechanical degradation processes, although more study is needed on the mechanisms involved. Degradation of PS products such as disposable plates, coffee cup lids, and foams has recently been simulated by using UV exposure (5) or by mechanical stress in the marine environment (6). Ekvall et al. (7) observed that the mechanical degradation of expanded foam as well as coffee cups and lids could produce small PS particles (MPs as well as NPs) (6).

The relatively easy absorption of these MPs and NPs from the gastrointestinal (GI) tract makes them a matter of concern (2, 8). MPs as well as NPs may be incorporated into many food products due to their widespread distribution and broad bioavailability in most terrestrial and aquatic environments. According to reports, there are a variety of ways that plastic MPs as well as NPs can be introduced into the human food chain, including the consumption of various food products obtained from exposed livestock, or the various stages of food processing involving plastic wrapping and packaging (9–11). Different foods (like honey or sea food) as well as beverages (like beer) have been found to be contaminated by MPs as well as NPs (12–16).

The routes by which these particles enter the body involve the mouth, esophagus, stomach, and intestines. This means the initial toxic effects are often observed in the GI tract. Some of the reported side effects include, disruption of the normal intestinal flora, decreased intestinal mucosal secretion, damage to the intestinal mucosal epithelium, and impaired metabolism of fatty acids and amino acids leading to the excessive accumulation of lipids (17). The chronic ingestion of MPs and NPs can negatively affect the gut barrier function. Previous studies reported that MPs and NPs could induce microbial dysbiosis in the gut (18, 19), or nose and lungs (20). NPs increased mRNA and protein levels of interleukin (IL)-8, IL-10, IL-1β and TNF-α in the gut of zebrafish to a greater extent than MPs, indicating that the NPs may have a more serious effect on gut microbial dysbiosis and inflammation (19). Demonstrated that oral administration of these MPs and NPs decreased beneficial intestinal microbes with known tight junction-promoting functionality, suggesting an important indirect toxic effect on the gut microbiota as well as NP-induced gut barrier dysfunction. In a mouse study, showed that MPs had a stronger effect on microbial dysbiosis in the lungs compared to NPs (18). Abnormal levels of *Staphylococcus* spp. in the nose, as well as *Roseburia*, *Eggerthella* and *Corynebacterium spp.* in the lungs were observed in both MP and NP exposed mice. These bacteria could be potential biomarkers of MP and NP-induced airway dysbiosis in mice (20).

The permissible range for chronic exposure to styrene has been set at 300 ppm (i.e., 1,000 mg/m^3^) by the Environmental Protection Agency (EPA). The FDA stated that the admissible daily intake (ADI) of styrene should be limited to 90,000 micrograms per person per day (4, 21).

One study developed a probabilistic model of lifelong exposure to plastic MPs for children and adults. In this physiology-based pharmacokinetic sub-model, the routes of administration were considered as intestinal absorption from food or by inhalation, while biliary excretion, and exposure to plastic-related chemicals was also included. The goal of this model was to simulate the concentration of MPs in the intestine, feces, and body tissues, while the stool samples provided validation versus empirical data. The median dose of MPs was calculated to be 583 ng per person per day in adults, and 184 ng per person per day in children. This dose could result in irreversible accumulation of particles in body tissues up to 6.4 ng per person for children under 18 years of age, and up to 40.7 ng per person for adults up to 70 years of age. They confirmed the agreement between the concentrations of the simulated MPs in the feces with the empirical data. The final analysis found there was a small share of MPs within the total chemical intake due to adsorption from food or MPs swallowed from 9 consumption simulations based on two-phase adsorption kinetics, reversibility, and specific size (22).

**Figure 1:**
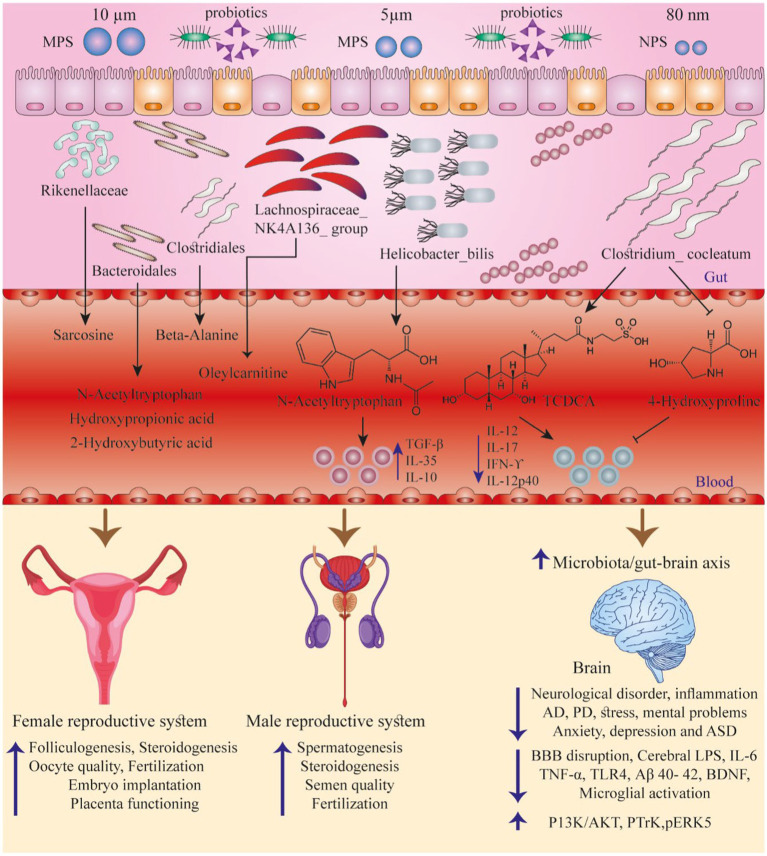
Summarizes some studies discussing the interactions between PS particles, GI normal flora, and the function of various organs. Adapted from (23).

The goal of the current review was therefore to summarize the absorption routes of PS MPs as well as NPs contaminating the food chain, their existing levels in foods, and possible side effects on human health. We further survey how probiotics could function to protect against these adverse effects in humans, and finally provide relevant suggestions for future work (Figure 1).

## 2. Literature searching and data acquisition

In this review, we searched for reports of the toxic hazard effects of PS MPs or NPs *in vitro* and *in vivo*. In addition, we investigated the detoxification activity of probiotics on chemical food contaminants, with an emphasis on the protective effects against PS MPs and NPs. We searched the electronic databases Web of Science, PubMed, EMBASE, CINAHL and Google Scholar from 2015 to 2023. The inclusion criteria were as follows: studies on the toxicity of PS MPs and NPs in various cell and animal models; protective effects of probiotic bacteria; suggested mechanisms of action. The exclusion criteria were as follows: studies written in a language other than English; studies did not focus on the toxicity of PS MPs and NPs; studies that did not discuss the relationship between the protective effects of probiotic bacteria against chemical contaminants. Considering that there was no study discussing the protective effect of probiotics specifically against the toxicity of PS MPs and NPs, several studies on the mechanism of detoxification by probiotics on various chemical food contaminants were included.

## 3. Oral ingestion, cellular internalization and diffusion of PS MPs and NPs

Oral ingestion of PS MPs as well as NPs has been identified as the main way these particles enter the human body (24). We found a few studies that examined the toxicity of PS NPs in humans, even though research has shown that plastic MPs are regularly ingested via drink and food products by the majority of the population in advanced countries (10, 25). Another study evaluated human colectomy samples for the presence of MPs, and detected the presence of MPs in all 11 samples with a mean value of 331 particles per sample or 28.1 ± 15.4 particles per gram of tissue, showing that MPs were ubiquitous within the human colon (26).

There is a very low probability of paracellular penetration of the MPs in the intestine, because the size of the biological pores in the narrow junction channels is only about 1.5 nm (27). NPs probably penetrate via the lymphatic tissue and in particular enter the microfold (M) cells of Peyer’s patches via phagocytosis (28). In one study, peritoneal macrophages in mice were able to phagocytose polystyrene and polymethacrylate particles of 1, 5, and 12 μm in size. They also confirmed the uptake of only 0.04–0.3% of these particles from the intestines of exposed mice (29, 30).

One important question is whether the NPs can penetrate the intestinal epithelium, which could result in systemic exposure in humans. Multiple *in vivo* as well as *in vitro* studies have been performed to investigate how PS NPs behave in a variety of animal models. Credible evidence has shown that when consumed orally, PS NPs may be 10 to 100 times more bioavailable than MPs (31, 32). Whether or not there is a correlation between absorption, size, and the structure of NPs is still controversial (1, 32).

Wang et al. (33) investigated the toxicity of PS NPs on the renal tubular epithelial cell line HKC and the human liver cell line HL-7702 using integrated proteomic and metabolomic analysis. Most of the differentially expressed proteins and metabolites were involved in a variety of metabolic pathways, for example, glycolysis, citrate cycle, oxidative phosphorylation, and amino acid metabolism, suggesting that PS NPs could disturb the global metabolism in human cells. The altered energy metabolism induced by PS NPs was confirmed by further studies. Moreover, mTORC1 signaling, a central regulator of cellular metabolism, was inhibited after nanoplastic exposure, likely serving as a link between lysosome dysfunction and metabolic disturbances (33). Cortés et al. (34) showed that PS NPs had toxic effects on human intestinal epithelial Caco-2 cells. According to their results, after the cells were exposed to PS NPs, cytotoxicity and ROS were increased, along with genotoxicity mediated by DNA oxidative damage, and an increase in the expression of stress-related genes.

Moreover when the PS NPs are swallowed and reach the gastrointestinal lumen, they can be mechanically or chemically degraded thus altering their rate of adsorption. The interaction of nanoparticles with different molecules such as carbohydrates, proteins, water, lipids, nucleic acids and ions within the GI tract according to the (35) may produce NPs that are surrounded by a set of proteins called a “protein corona” (36). The PS NPs may then become coated with protein coronas with different complex shapes or chemical properties (37). Walczak et al. (32) showed that the protein-based corona underwent changes when subjected to a laboratory model of human digestive fluid, resulting in further morphological variation in the NPs. Therefore, ingested PS NPs, depending on their size and physicochemical properties, could potentially translocate across the intestinal barrier (32). Furthermore, the NPs may interact with dispersed organic matter or metal nanoparticles present in the marine environment, which could then affect their aggregation and deposition in the ocean, and thus their uptake by various types of seafood (37).

Active endocytosis and passive diffusion are the two main mechanisms by which plastic particles can be taken up into cells (38–40). Endocytosis includes phagocytosis and pinocytosis, e.g., clathrin-mediated macropinocytosis, caveolin-mediated pinocytosis, as well as pinocytosis independent of clathrin/caveolin (41). Macrophages, neutrophils, monocytes, and dendritic cells are designed to carry out phagocytosis, i.e., professional phagocytes (42). Endothelial cells may internalize 40 nm PS NPs through caveolin-mediated as well as clathrin-mediated endocytosis (43). Reportedly, the endocytosis of PS particles with different sizes of 20, 120, 190, and 200 nm occurred via the clathrin-mediated pathway in canine renal cells, lung epithelial cells, and murine melanoma cells (44–46).

Another study reported that clathrin-mediated endocytosis was responsible for the uptake of particles smaller than 200 nm, while caveolin-mediated endocytosis occurred for particles bigger than 500 nm (46). However, porcine endothelial cells used macropinocytosis (independent of caveolae or clathrin) for the internalization of PS NPs smaller than 100 nm, (47), while Caco-2 cells (human colorectal carcinoma) adsorbed these particles by micropinocytosis (48). The MPs larger than 1 μm were not taken up by non-phagocytic eukaryotic cells through endocytosis, but were taken up by macrophages through micropinocytosis or phagocytosis (43, 46). Thus, the size of the PS particles and the cell type determine the type of endocytosis employed for cell uptake. The particle size may also be correlated with the rate of cell uptake, because the smaller the particle, the faster the rate of cell uptake (46).

Passive diffusion appears to be the pathway of choice for internalization of carboxylate as well as amidine-modified PS particles of 20 nm and 120 nm diameter into alveolar epithelial cells (49). The mechanisms of cellular excretion of these particles may also be divided into active and passive pathways (38, 50). If the particles enter the cell passively, they may then enter the lysosomes and undergo active exocytosis in an energy-consuming process, or may exit the cell passively without any energy expenditure. If the particles enter the cell through endocytosis they are automatically routed to lysosomes, and will be excreted from the cells by active exocytosis (51). In conclusion, the particular route of cell uptake for PS particles will also affect their excretion mechanisms.

## 4. Toxic effects of PS particles

Generally speaking, the cellular effects of PS particles will differ according to their size and concentration, as well as the type of organism or cells that are exposed to the PS particles, and the duration of exposure (2, 52). In the nervous system of mice, PS particles (MPs or NPs) may alter the level of various neurotransmitters (catecholamines, acetylcholine, and serotonin) which could then affect stress responses and behavior (52, 53). These PS particles could also increase the level of nitric oxide (NO), acetylcholine (by inhibiting acetylcholine esterase), and thiobarbituric acid reactive substances (TBARS) (52), which are a marker of oxidative damage (54). Increased acetylcholine and oxidative damage could explain the memory deficits observed in mice exposed to PS particles (54).

With reference to possible genotoxic effects, it was reported that when *Ctenopharyngodon idella* (grass carp) were exposed to PS NPs, DNA molecules were damaged either by strand breaks or cross-links with other molecules. The DNA damage could be caused either by direct interaction between the particles and the DNA, or by disturbances in cholesterol metabolism, which then produces free radicals (52).

Some evidence for PS MP-mediated mutagenesis was provided by the detection of nuclear contracted erythrocytes, dual-nucleated erythrocytes, kidney-shaped displaced nuclei, micronuclei, nuclear vacuoles and notched nuclei. In addition, cytotoxic effects were related to the MP shape and size (52). Mutagenicity as well as the formation of oxygen free radicals caused by PS NPs has also been reported in HCT116 colorectal cancer cells (55).

Mice with experimental colitis exposed to PS MPs showed an increased inflammatory response (56). The proinflammatory cytokine levels were significantly increased by PS MPs, which in turn could cause abnormal lipid metabolism and elevated triglyceride levels. These changes could promote monocyte recruitment into tissues and a stronger inflammatory response, i.e., formation of a vicious cycle (56, 57). The elevated triglycerides may be taken up by hepatocytes, resulting in hepatitis, or the PS MPs may directly find their way into the liver tissue to cause hepatitis (56).

Ding et al. treated mice with PS NPs for 3 days, and identified their presence in intestinal, gastric, as well as hepatic cells. In a mechanistic study, they used GES-1 cells (gastric epithelial cells), and found that PS NPs could increase the formation of reactive oxygen species (ROS), which in turn inhibited growth and caused apoptosis in these cells. They proposed that these particles could interfere with the barrier function of the epithelium in the GI tract (58).

Pan et al. treated mice with PS MPs for 90 days to assess their hepatotoxic effects. They found that PS MPs could activate PERK (protein kinase RNA-like endoplasmic reticulum kinase in murine hepatocytes to increase ER stress and induce apoptosis). The fact that PERK knockdown inhibited the induction of apoptosis confirmed this finding (59).

Murine models were also designed to evaluate the toxic effects of PS MPs in the germinal organs, i.e., ovaries (60) and testes (60, 61). These researchers observed that different cell types in these organs adsorbed the particles with a consequent increase in oxidative damage, which in turn activated signaling cascades, such as NLRP3/Caspase-I or Nrf2/HO-1/NF-kB (60) to induce apoptosis.

Shengchen et al. (62) examined how PS MPs would affect the injury response of murine rhabdomyocytes and their ability to repair muscle damage. Again, it was observed that these particles increased oxidative damage in the cells, and inhibited rhabdomyocyte proliferation, as well as their ability to repair the muscle tissue. Furthermore, the PS MPs prevented MAPK from being phosphorylated, while activating NF-κB, which induced the myocytes to differentiate into adipocytes.

In fish exposed to MPs, hematological parameters can be used as sensitive indicators (63). The hematopoietic system of mammals has also been studied, where it was found that PS NPs accumulated in the bone marrow of mice and altered cellular functions (31, 64). Mice treated with PS particles showed a lower number of white blood cells, but a higher number of thrombocytes (64, 65). *In vitro*, it was shown that PS NPs increased oxidative stress as well as DNA damage in human lymphocytes (66).

Table 1 lists some published studies on the toxicity of PS particles *in vivo* as well as *in vitro*.

**Table 1:** Studies discussing the cytotoxic effects of polystyrene (PS) particles.

**Study**	**Model**	**Sample size**	**Survey**	**Intervention**	**Duration**	**Result**
(20)	5 week-old male mice	12 mice in each group	Nasal and lung microbial dysbiosis	10 μL MP/NP suspension (containing 100 μg PS MP or PS NP)	5 weeks	Airborne PS MPs and PS NPs could alter the nasal microbiota in mice, and MPs had a stronger effect on the lung microbiota than NPs. Nasal *Staphylococcus*, lung *Roseburia*, lung *Eggerthella* and lung *Corynebacterium* were associated with both MPs and NPs, which could be biomarkers of MP and NP-induced airway dysbiosis in mice.
(33)	HKC (renal tubular epithelial cell line) HL-7702 (human derived liver cell line)	–	Cytotoxicity	80 nm diameter − 50 and 100 μg/mL PS NP	0.5 to 36 h	Most of the differential proteins and metabolites were enriched in various metabolic pathways, glycolysis, citrate cycle, oxidative phosphorylation, and amino acid metabolism, suggesting the potential effects of PS NPs on ga lobal cellular metabolic shift in human cells. Altered energy metabolism induced by PS NPs was further confirmed. mTORC1 signaling, a central regulator of cellular metabolism, was inhibited by nanoplastic exposure, likely serving as a link between lysosome dysfunction and metabolic defects.
(67)	Wild-type zebrafish	10 fish in each group	Immunotoxicity and effect on intestinal microbiota	50 μm diameter − 100 μg/mL PS MP 50 μm diameter − 1,000 μg/mL PS MP 100 nm diameter − 100 μg/mL PS NP 100 nm diameter − 1,000 μg/mL PS NP	14 days	PS particles caused damage to intestinal epithelium, altered the normal flora composition of GI tract, induced oxidative damage, and also interfered with immune responses.
(68)	Immature Crucian carp	10 fish in each group	Toxicity effects on gut microbiota	100 μg/L aged PS MPs 100 μg/L roxithromycin +100 μg/L aged PS MPs	28 days	Aging of PS MPs enhanced their binding to roxithromycin, and the combined administration of these two substances had a more pronounced effect on inducing inflammation as well as inhibition of amylase and lipase; the combined administration also altered the composition of normal flora of GI tract; aged PS MPs were also able to induce antibiotic resistance in gut microbiota.
(23)	4 week-old C57BL/6 J male mice	7 mice in each group	Hematopoietic damages	PS MPs (5 μm and 10 μm) as well as PS NPs (80 nm) at a concentration of 60 μg/day	42 days	Alterations in serum cytokines were detected which may be explained by dysbiosis, and pathologic changes in the bone marrow, i.e., elevation of lipids and inhibition of differentiation of hematopoietic stem cells.
(69)	6 week-old male C57-BL/6 mice	8 mice in each group	Intestinal immune imbalance	PS MPs (500 μg/L)	28 days	PS MPs caused a more pronounced inflammatory response (local and systemic) in mice with dysbiotis compared to normal flora, and also aggravated the dysbiosis.
(68)	Nile tilapia (*Oreochromis niloticus*)	40 fish in each group	Immunotoxicity and disturbance of intestinal microbiota	PS MPs (1 mg/L) alone or in combination with Cu^2+^ (0.5, 1, and 2 mg/L)	14 days	PS MPs aggravated the toxic effects of Cu^2+^ including: hepatic infiltration, pathologic changes in hepatic, intestinal, and gill tissues, oxidative damage, impaired immunity, as well as dysbiosis.
(33)	Juvenile grouper	40 fish in each group	Toxic effects on digestive system	PS NPs (300 and 3,000 μg/L)	14 days	PS NPs accumulated in the liver and intestinal tissues, weakened the digestive ability of the GI tract, induced dysbiosis, and hampered the growth of the fish.
(70)	Zebrafish	20 fish in each group	Effects on the intestinal tissue and its normal flora	1 mg/L of commercial MPs (CMPs) or realistic MPs (RMPs) given alone or in combination with 0.5 μg/L enrofloxacin	28 days	Dysbiosis caused by CMPs was more severe than RMPs, and they both induced resistance to enrofloxacin in th egut microbiota.
(52)	Swiss mice	12 mice in each group	Neurotoxic, biochemical and genotoxic effects	PS NPs (14.6 ng/kg) Intraperitoneal administration	3 days	PS NPs accumulated in the brain resulting in elevated levels of NO, thiobarbituric acid reactive species, and acetylcholine, which impaired cognitive function of the mice; DNA damage in erythrocytes and hyperlipidemia were also observed.
(71)	*Ctenopharyngodon idella* (grass carp) juveniles	21 fish in each group	Biochemical, genotoxic, mutagenic and cytotoxic effects	PS NPs (-I group 0.04 and 34 ng/L, 34 μg/L)	20 days	PS NPs caused mutagenic effects, oxidative damage, and morphological changes in erythrocytes; infiltration into hepatic and brain tissues was also observed.
(56)	6 week old male C57 mice	Total of 50 mice	Biochemical, metabolomics and histopathological analysis of the liver	PS MPs (5 μm, 500 μg/L) Oral administration (in distilled water)	28 days	PS MPs caused a proinflammatory response as well as altered metabolic activity of the liver in mice, whether normal or with colitis; the elevated fat content of hepatocytes was only significant in mice with colitis.
(59)	8 week old male C57BL/6 J mice	12 mice ineach group	Hepatotoxicity	PS MPs (5 μm, 0.1 mg/day) Oral administration (in distilled water)	90 days	Increased ER stress in hepatocytes by PERK activation.
(60)	6 week old female Wistar rats	8 mice in each group	Ovarian toxicity	PS MPs (0.5 μm) at doses of 0.015, 0.15 and 1.5 mg/kg/d Oral administration (in distilled water)	90 days	Increased oxidative stress in granulosa cells resulting in more apoptosis, and fewer ovarian follicles.
(60)	4–5 week old ICR male mice	10 mice in each group	Testicular toxicity	PS MPs (5 μm) at concentrations of 100 and 1,000 μg/L, and 10 mg/L Oral administration (in distilled water)	35 days	Testicular sperm showed an accelerated apoptotic rate due to increased inflammatory cytokines in testicular tissue. Epididymal sperm showed a decreased number and more dysmorphic changes.
(62)	6 week old C57BL/6 male mice	10 mice in each group	Skeletal muscle toxicity	PS MPs (10 mg/L) at two size ranges: 1–10 μm and 50–100 μm	30 days	Increased oxidative damage inhibited the rhabdomyocytes ability to proliferate and repair the muscle tissue. Furthermore, PS MPs prevented MAPK from being phosphorylated, while activating NF-κB to drive myocyte differentiation into adipocytes.
(58)	5 week old C57BL/6 male mice GES-1 cells	6 mice in each group	Cytotoxicity	PS NPs at a concentration of 50 μg/mL Oral gavage (in double distilled water)	3 days	In mice, PS NPs infiltrated into gastric, intestinal, as well as hepatic tissue PS NPs were endocytosed into GES-1 cells *in vitro* to induce apoptosis.
(72)	6 week old C57BL/6 J mice	15 mice in each group in the 24 h experiment 10, 15, or 25 mice in each group in the 28 day experiment	Intestinal barrier dysfunction	PS particles with three sizes: 50, 500, and 5,000 nm; dose range of 2.5 to 500 mg/kg/day	24 h 28 days	In 24 h experiment: the PS NPs and MPs infiltrated into intestinal epithelial cells, infiltration was higher when NPs and MPs were co-administered; infiltration induced apoptosis in intestinal epithelial cells to interfere with their barrier function; infiltration of PS particles was also detected in various other tissues. The 28 day experiment: confirmed the disruption of barrier function.
(66)	Raji-B and TK6 cells (lymphocytic cell lines) THP-1 cells (monocytic cell line)	–	Human hematopoietic cell toxicity	PS NPs about 50 nm size and a concentration range up to 200 μg/mL	3 to 48 h	The monocytic cell line internalized the more NPs, but the oxidative and mutagenic effects were not significant; the lymphocytic cell lines internalized fewer NPS, but the toxic effects were more pronounced.
(73)	Caco-2 cells	–	Intestinal toxicity	PS particles and transformed PS particles with two sizes: 5 and 100 nm, and two concentrations: 1 and 20 μg/mL	96 h	Model digestive fluid (designed to simulate the human GI tract) was added to PS particles, which developed a corona on their surface, but did not alter their chemical composition; these corona PS particles were called transformed. This transformation reduced the oxidative damage and penetration of particles through the epithelial layer, but promoted the inflammatory response.
(74)	Single cell organisms: bacteria (*E. coli, S. aureus, V. fischeri*), yeast (*S. cerevisiae* wild type and end3Δ strains), algae (*Raphidocelis subcapitata*), and protozoa (*Tetrahymena thermophila*) Multicellular oraganisms: *Daphnia magna, Heterocypris incongruens, and Chironomus riparius* THP-1 cells (derived from human monocytes)	–	Cytotoxicity	Commercial PS NPs with two sizes: 26 and 100 nm, and doses up to 100 mg/L	30 min to 6 days	Cytotoxic effects were only observed in *V. fischeri, R. subcapitata, and D. magna*; further evaluation showed that even this toxicity may not be caused by the PS NPs, but may be partially attributed to NaN_3_, a cytotoxic additive present in commercial preparations of PS NPs.
(75)	HepG2 cells	–	Cytotoxicity	PS NPs (50 nm) at three doses: 10, 50, and 100 μg/mL	24 h	PS NPs with carboxyl and amine groups on their surface induced more oxidative damage.
(76)	Human peripheral blood mononuclear cells KATO III cells HeLa cells Human dermal fibroblasts	–	Cytotoxicity	PS particles with random shapes from 5 to 200 μm size, three different concentrations: 10, 100, and 1,000 μg/mL	1 and 4 days	Oxidative damage in all cell lines; these particles were also able to physically rupture the cellular membrane.
(77)	5–6 week-old Balb/c male mice	10 mice in each group	Reproductive toxicity	PS MPs (5.0–5.9 μm) at three different doses: 0.01, 0.1, and 1 mg/day N-acetylcysteine (anti-oxidant) and SB203580 (inhibitor of MAPK) were also co-administered Oral gavage	42 days	PS MPs induced oxidative damage in testicular tissue through MAPK activation, which resulted in reduced sperm numbers, which were dysmotile and dysmorphic;,the testosterone level in plasma was also decreased; administration of N-acetylcysteine or SB203580 was able to alleviate these effects.
(78)	Human mast cell line1 Peripheral blood mononuclear cells Human dermal fibroblasts Sheep RBCs	–	Cytotoxicity	PS particles with six diameters: 460 nm, 1 μm, 3 μm, 10 μm, 40 μm, and 100 μm, and doses of 1, 10, 100, 500, and 1,000 μg/mL	–	In general, the PS particles did not cause notable cytotoxicity in human cell lines, but could damage RBCs through direct contact; inflammatory cytokine profile was also altered.
(79)	*Mus musculus* mice	5 mice in each group	Biodistribution	PS particles with two diameters: 5 and 20 μm, and three doses: 0.01, 0.1, and 0.5 mg/day. Oral administration	28 days	PS particles accumulated in intestinal, hepatic, and renal tissues with subsequent oxidative damage, which may be affected by the particle size.
(80)	Caco-2, HT29, Raji B, and THP-1 cell lines Male Hmox1 reporter mice	5 mice in each group	Cytotoxicity	1, 4, and 10 μm PS particles at doses of (4.55 × 10^7^ particles), (4.55 × 10^7^ particles) and (1.49 × 10^6^ particles), respectively	*In vitro* study: 24 or 48 h *In vivo* study: 28 days	Neither *in vitro* nor *in vivo* studies showed notable cytotoxic effects attributable to PS particles; the ability of THP-1 cells to differentiate into different types of macrophages was also preserved.
(81)	6–8 week-old male Wistar rats	6 rats in each group	Neurobehavioral assessment	PS particles with two diameters: 25 and 50 nm, and doses of 1, 3, 6, and 10 mg /kg /day Oral administration	35 days	No notable neurobehavioral dysfunction.
(82)	Oysters	40 oysters per tank	Reproductive toxicity	2 and 6 μm PS MPs at a concentration of 0.023 mg/L	60 days	Reduced number of oocytes and motility of sperm; developmental progress of offspring was slower.
(83)	AGS gastric cancer cell line	–	Cytotoxicity	PS particles with diameters of 44 nm and 100 nm and doses of 1, 2, and 10 μg/mL	1 and 24 h	Both NP sizes accumulated in gastric cancer cells; the effects on cellular viability were paradoxical; but they both upregulated IL-6 and IL-8.

## 5. Probiotics and their protective effects

Probiotics are living microbial supplements which can be consumed by the host. They have beneficial effects on the host by promoting a better microbial balance within the gut and by modulating the host immune system (84–87). These microorganisms, specifically lactic acid bacteria (LAB), have been reported to control hypertension, modify lipid profiles and hyperglycemia, and also suppress oxidative damage (88, 89).

Probiotic microorganisms, whether they be the normal flora of the GI tract, or are consumed as microbial supplements may also interact with PS particles to modify their toxic effects on different tissues (90, 91). With regard to the hematopoietic system, a relationship between the intestinal microflora and hematological disorders has been observed in mammals (92). Excessive antibiotic administration can paradoxically cause bacterial infections in the gut (*Clostridium difficile*), which can also cause neutropenia, thrombocytopenia, and pancytopenia, demonstrating how various microorganisms can affect the interactions between the gut and the hematopoietic system (93, 94). However, the precise mechanisms explaining why probiotics could modify PS hematotoxicity remain elusive (23). One probable explanation may be that probiotics can produce specific small molecule metabolites, or else drive signaling cascades, which help the organism to boost its immune response or to suppress inappropriate inflammatory responses (95–98).

### 5.1. Mechanism of the protective effects of probiotics against damage by toxic materials

The introduction of probiotic bacteria (including LAB strains) into the GI tract can eliminate or decrease the toxic effects of heavy metals or toxic fungi (99). Bacterial molecules like mannan oligosaccharides or peptidoglycans present in the cell wall of these organisms can enable the cells to bind to these toxins (99–101). Some probiotic bacteria and yeasts, including *L. rhamnosus*, *L. plantarum*, and *Saccharomyces cerevisiae*, are able to adsorb various heavy metals like lead, cadmium, copper, as well as mercury (102–104). Probiotics can also bind to molecules and toxins like benzo[a]pyrene (105), mycotoxins (106), bisphenol A (BPA), or phthalates (100).

Some probiotics were even able to degrade the common plastic ingredient, bisphenol A (107, 108). Given the ability of these microorganisms to affect the toxicodynamics of different endocrine-disrupting chemicals, the use of probiotic supplementation for improving the microbiome could be an effective intervention to counter different toxins (100, 109). In diabetic patients, it was observed that probiotics could decrease the level of systemic inflammation (110) and also control hyperglycemia (88, 111). It should be noted that compared to single-strain probiotics, polybiotics (mixed probiotics) have a greater ability to bind to toxins (112, 113). Another factor that may affect the toxin-binding ability of the probiotics is the pH of the GI tract. For example, the biosorptive ability of *L. plantarum* P1 was observed to be higher in an alkaline environment, probably because the cell wall structure is more stable at a higher pH (114).

The natural gut microbiota is composed of beneficial as well as pathogenic bacterial species, and when the pathogenic species dominate over the beneficial species, this is called gut dysbiosis. Dysbiosis in the gut has been associated with worse gastrointestinal damage after oral exposure to MPs and NPs (18, 115). Microbial dysbiosis leads to a more pronounced inflammatory immune response, increases oxidative damage, and stimulates the progression of cancer (116). Several studies have reported the use of probiotic strains to modulate the gut microbiome and normalize immune responses for the prevention and management of intestinal dysbiosis (Table 2).

**Table 2:** Studies discussing the detoxification activity of probiotics.

**Study**	**Model**	**Activity**	**Probiotics**	**Biological effects (host’s response)**
(117)	Male Sprague Dawley rats	Aflatoxin B1 (AFB1) detoxification in bowel	*Lactobacillus casei Shirota*	The cytotoxic effects, the inflammation in intestinal mucosa, and the dysbiosis caused by AFB1 were all relieved by *L. casei*.
(118)	NCM460 Intestinal epithelial cells	Lycopene production and intestinal oxidative damage	*Lactococcus lactis*	Increased lycopene accumulation. Protected intestinal epithelial cells against H2O2 challenge.
(119)	Interactions between probiotics and benzo[a]pyrene in phosphate buffered saline	Detoxification of of benzo[a]pyrene	*Lactobacillus rhamnosus* *Bifidobacterium lactis* *Lactobacillus casei* *Lactobacillus acidophilus* *Lactobacillus delbrueckii* *Lactobacillus casei* *Lactobacillus brevis* *Streptococcus thermophilus*	Different strains had different ability to bind to and inhibit benzo[a]pyrene.
(120)	*Liza ramada* (thinlip mullet)	AFB1 detoxification	*Lactobacillus acidophilus*	*L. acidophilus* protected hepatic, renal, and hematopoietic systems against AFB1 damage; induced a healthy lipid profile; and controlled hyperglycemia and oxidative damage
(100)	Male albino rats	Phthalates and bisphenol A (BPA) detoxification	*Saccharomyces boulardii* *Lactobacillus rhamnosus* *Lactobacillus plantarum*	Probiotics were able to reduce apoptosis and oxidative damage caused by BPA and phthalates in the pancreas.
(121)	Interactions between phthalate and probiotics after they were co-incubated	Phthalate detoxification	*Lactobacillus plantarum* CGMCC18980	*L. plantarum* CGMCC18980 could potently bind to phthalate molecules mainly mediated by the exopolysaccharide present in the cell walls.
(122)	Caco-2 cell line	Phthalate detoxification	*Lactobacillus acidophilus* NCFM	*L. acidophilus* NCFM inhibited the toxicity of phthalate both by binding to it, and by modifying the signaling pathways which mediate phthalate toxicity.
(123)	Wistar albino rats	Dichlorodiphenyltrichloroethane detoxification	*Bifidobacterium infantis* *Lactobacillus acidophilus* *Bifidobacterium thermophilum* *Lactobacillus casei* *Bifidobacterium longum* *Lactobacillus helveticus Lactobacillus plantarum Lactococcus lactis* *Leuconostoc mesenteroides* *Lactobacillus paracasei* *Lactobacillus brevis*	Probiotics lowered systemic inflammation and inhibited oxidative damage in hepatic and splenic tissues.
(114)	Male SPF rats *In vitro* binding of the probiotic and the toxin	Phthalate detoxification	Eight strains of Lactobacillus	The binding capacity of these Lactobacilli strains, especially *L. planarum* P1, to phthalate was observed both *in vitro* and *in vivo*.
(109)	Wistar rats	Phthalates and bisphenol A (BPA) detoxification	*Saccharomyces boulardii* and three strains of Lactobacillus	Beneficial effects on endocrine system, hepatic, renal, and splenic tissues; plus anti-oxidant properties at the cellular level.

Clinical trials have also highlighted the efficacy of probiotic strains in reducing the side effects of microbial dysbiosis caused by various diseases or medical treatments (124–126). Showed that consumption of a complex mixture of probiotics (*Bifidobacterium infantis*, *Lactobacillus acidophilus*, *Enterococcus faecalis*, and *Bacillus cereus*) significantly restored the gut and oral microbial diversity(125). Probiotics comsumption increased the prevalence of *Holdemanella*, *Enterococcus*, and *Coprococcus_2* species, while it decreased *Fusobacterium*, *Eubacterium_ruminantium*_group, *Ruminococcus_1*, and *Parasutterella* in the human gut. The use of probiotics can improve the gut microbial population, increase mucus-secretion, and prevent the destruction of tight junction proteins by decreasing the level of lipopolysaccharides (LPS). Furthermore, the decrease in gut dysbiosis and intestinal leakage after probiotics therapy could reduce the secretion of inflammatory biomarkers and blunt any unnecessary activation of the immune system (127).

### 5.2. Mechanism of the protective effects of probiotics against organ damage

The consumption of beneficial probiotics can affect body weight, glucose and lipid metabolism, resulting in less systemic inflammation as well as improved insulin sensitivity (100, 128).

Baralić et al. treated mice with a combination of BPA and phthalates (toxins often occurring in the human daily diet) and simultaneously administered polybiotics (mainly different species of Lactobacillus) to examine how these probiotics would modify the toxicity of these compounds. The probiotics showed a beneficial effect on the endocrine system, as well as hepatic, renal, and splenic tissues. Furthermore, it was reported that at the cellular level probiotics may have anti-oxidant properties (100).

With regard to the biosorptive ability or toxin binding, this ability of probiotics has been repeatedly confirmed by different studies, and was found to be due to the binding properties of specific protein and polysaccharide structures present in their cell walls (121, 129–131).

With regard to hepatoprotective effects*, S. boulardii* was able to improve liver function as well reduce liver damage caused by hepatic steatosis, liver fibrosis, or infections (132). As mentioned above, the hepatoprotective effects of probiotics were discussed by Baralić et al. (109).

*Lactobacillus* spp. and *S. boulardii* can act as antioxidants by preventing lipids from being peroxidized, as well as by activating antioxidant enzymes (133). *S. boulardii* was able to improve the tissue antioxidant status and inhibit neutrophil recruitment, thereby reducing intestinal damage and associated hepatitis (134). A reduction of oxidative damage in hepatocytes by probiotics was also reported by Oumeddour et al. (135).

In another study, mice received oral *L. rhamnosus* GG over 13 weeks, after which they were protected from insulin resistance as well as showing less adiposity in mesenteric adipose tissue and the liver. However, they also found increased fatty acid oxidation in muscles and liver, a reduction in liver gluconeogenesis, increased muscle expression of GLUT4, and increased white adipose tissue secretion of adiponectin (136). There was a reduction in hepatic total lipid levels and lower liver weight in mice with leptin-resistant obesity and type 2 diabetes, following daily oral gavage over 4 weeks with *S. boulardii* (137).

A murine model of colitis was employed by Kim et al. to test the effects of *L. plantarum* CBT. They found systemic anti-inflammatory effects and an improved immunomodulatory function, predominantly in lymphocytes (138).

The kidney protective effects of probiotics were reported in a study by Baralić et al. (109). They found a lower serum urea level as well as less nephronic damage in pathologic assessments of tissue from mice treated with probiotics (109). The nephroprotective effects of these microorganisms against mercury toxicity were also reported by Majlesi et al. (139).

### 5.3. Probiotics and neurotoxicity

In a study by Alipour Nosrani et al. (140) polybiotics (consisting of *B. bifidum*, *L. acidophilus*, *L. fermentum* and *L. reuteri*) were administered to patients with Parkinson’s disease. They found improvements in cognitive function, rotational motor responses, less nerve injury and lipid peroxidation.

Cheon et al. cultured SH-SY5Y cells (neuroblastoma cell line) to assess the neuroprotective effects of three strains of *Lactobacillus.* MPP+ (1-methyl-4-phenylpyridinium) was used as a neuotoxin to induce Parkinson’s disease-like neuronal damage. In general, the probiotic treatment was able to inhibit cellular death induced by MPP+, and at the molecular level, it upregulated brain-derived neurotrophic factor (BDNF) (141).

### 5.4. Probiotics and reproductive toxicity

One study combined probiotics (*L. salivarius*, *L. brevis*, *L. planarum*) in the form of a human vaginal tablet to treat bacterial vaginosis. They also investigated the protective effects of this probiotic preparation on the peroxidation of sperm lipids, which resulted in maintainance of sperm vitality and motility (142). Dardmeh et al. (143) recently suggested that probiotics could be an option to reduce the adverse consequences of obesity on the quality of semen. The effect of probiotic *L. rhamnosus* was evaluated on lipid status, testicular weight, sperm kinetics and relevant hormones like LH, FSH, as well as testosterone in mice. The direct effect was to improve sperm maturation and spermatogenesis, and the indirect effect was to eliminate obesity-related complications.

Another recent study examined the effect of probiotics on relieving the inflammation caused by polycystic ovary syndrome (PCOS). PCOS is a treatable endocrine disorder, associated with ovarian dysfunction, excessive secretion of testosterone, increased inflammation, and consequent infertility. The beneficial effects of *Lactobacillus* in PCOS patients, included reduction of inflammation, as demonstrated by lower IL-6 and higher IL-10 levels, which resulted in improved fertility (144).

### 5.5. Mechanism of protective effects of probiotics against mutagenicity and cytotoxicity

The carcinogenicity and genotoxicity of heterocyclic aromatic amines could be decreased by probiotics, owing to their ability to bind to toxins (122). One study demonstrated that *L. rhamnosus* GG was able to absorb 61.0% of aflatoxin B1 (AFB1) administered to Caco-2 cells, after one hour of incubation at 37°C. The cells exposed to AFB1 alone underwent DNA fragmentation, but not when co-incubated with the probiotic strain (145). In the human hepatoma cell line (HepG2), there was a significant decrease in the generation of micronuclei mediated by patulin and ochratoxin A in the presence of *B. animalis* VM12 as well as *L. acidophilus* VM20 (146). In another study using Caco-2 cells, *L. acidophilus* NCFM was able to inhibit DNA damage and cell death induced by phthalate, mainly by altering expression levels of genes related to P53, MAPK and mTOR pathways (122).

## 6. Conclusion

The broad use of plastics and their products are steadily increasing today. Due to various benefits, such as low cost and suitable physicochemical properties, plastics are being used in many industrial products involved in construction, packaging, and transport. This wide use of plastics has led to the contamination of all possible natural environments, i.e., water, soil, and air, with microplastic or nanoplastic particles as well as bulk plastic. These plastics (especially PS) undergo degradation in these environments to produce particles with micro and nano sizes, which may find their way into the body of different organisms, including humans. They have been proved to have toxic effects on many different lifeforms. Hence, finding or developing new platforms/procedures for reducing the toxicity of nanoplastics and microplastics are needed.

According to studies on toxicity effects of MPS/NPS on gut microbiota, we suggest that major mechanisms that may be recruited by probiotics against MPS/NPS, are enhancement of the intestinal epithelial barrier, production of various cytokines and chemokines from dendritic cells, lymphocytes, macrophages, mast cells, granulocytes, and intestinal epithelial cells, and IgA-producing cells and consequent IgA secretion, colonization and normalization of perturbed intestinal microbial communities (147, 148). In addition, probiotics production of volatile fatty acids, namely, short-chain fatty acids and branched-chain fatty acids, increase of anti-inflammatory effects, e.g., through alterations of TLR4/TLR2/MyD88/NF𝂻 signaling and alterations in the NLRP3 inflammasome, which play a role in the maintenance of energy homeostasis and regulation of functionality in peripheral tissues. Probiotic metabolites are also able to interact with the brain-gut axis and enhancement of the CNS homeostasis. In this regard, it has been suggested that the use of probiotics might reduce the toxicity of these materials in humans; a claim that needs further evaluation.

## Author contributions

JB and ZB: investigation, validation, writing-review, and editing. RS: investigation, project administration, validation, writing-original draft, writing-review, and editing. MH: writing-review and editing. All authors contributed to the article and approved the submitted version.

## Conflict of interest

The authors declare that the research was conducted in the absence of any commercial or financial relationships that could be construed as a potential conflict of interest.

## Publisher’s note

All claims expressed in this article are solely those of the authors and do not necessarily represent those of their affiliated organizations, or those of the publisher, the editors and the reviewers. Any product that may be evaluated in this article, or claim that may be made by its manufacturer, is not guaranteed or endorsed by the publisher.
